# Promoting salutogenic pathways to health through complementary and integrative health approaches

**DOI:** 10.3389/fpsyg.2024.1473735

**Published:** 2024-10-02

**Authors:** Erin Burke Quinlan, Jennifer Baumgartner, Wen G. Chen, Wendy Weber, Emrin Horgusluoglu, Emmeline Edwards

**Affiliations:** Division of Extramural Research, National Center for Complementary and Integrative Health, National Institutes of Health, Bethesda, MD, United States

**Keywords:** salutogenesis, stress, resilience, whole person health, emotional well-being, complementary and integrative health

## Abstract

Health restoration and disease prevention are important strategies to achieve health and well-being. This *Perspective* provides a conceptual overview of the key concepts of salutogenesis (health restoration), chronic stress, resilience, and emotional well-being, and describes how they are distinct and interrelated. We posit, and demonstrate through scientific evidence, that complementary and integrative health approaches, including mind and body interventions, can be used to mitigate the effects of chronic stress and promote salutogenic pathways. Our goal is to identify research gaps and opportunities and suggest ways to advance the knowledge base for mechanistic and clinical research in this field.

## Introduction

Health and disease are not separate, disconnected states. Rather, they exist on a continuum, wherein a person can move toward or away from a state of health. The process by which individuals travel along this continuum from a less healthy state to a healthier state can be referred to as salutogenesis or health restoration (Antonovsky, 1979; Mittelmark and Bauer, 2017). The concept of salutogenesis is intricately linked to the stress process (Lazarus and Folkman, 1984), in that an acute stressor can be adaptive in short bursts and can even engage restorative processes such as improving cellular resistance to damage and growing new neural pathways. Conversely, chronic stress, burnout, and depression have the potential to push people toward a state of disease and accelerate aging (Yegorov et al., 2020).

The National Institutes of Health (NIH) Trans-NIH Resilience Working Group defines resilience as the “ability to resist, recover, adapt, or grow from a challenge” (Brown et al., 2023). Evidence-based research has established that resilience to stress is crucial to the health and well-being of individuals and of society. Resilience occurs over both long-and short-time scales and likely emerges from interactions across circuits that span molecules to the whole person through interconnected systems (Holz et al., 2020). However, if stressor exposure is chronic and/or severe, or occurs during a vulnerable period (e.g., childhood), then adaptive neurobiological and behavioral responses to stressors and potential for future resilience may be diminished for some individuals without intervention (Liu et al., 2018; Lupien et al., 2018; McEwen, 2016a). Multiple pathways and mechanisms modulate the complex interactions among stress, salutogenesis, resilience, health, and emotional well-being (EWB) (Table 1). For example, a sense of coherence plays a role in the interactions among, stress, resilience, and health (McGee et al., 2018), and stress-integrative brain regions shape the neural architecture underlying individual differences in susceptibility and resilience to chronic stress (Anacker et al., 2016; Isingrini et al., 2016). Chronic stress also plays a central role in emotional experiences of daily life and engages critical psychological defense mechanisms such as overall life satisfaction, social connectedness, sense of meaning, and purpose to mitigate the deleterious effects of chronic stress (Esch et al., 2024; Scott et al., 2013; Sliwinski et al., 2021).

**Table 1:** Operational definitions of health, salutogenesis, stress, resilience, and emotional well-being.

Term	Definition
Health	A state of physical, mental, and social well-being and not merely the absence of disease and infirmity (Centers for Disease Control and Prevention, n.d.).
Salutogenesis	The process by which individuals move from a less healthy state to a healthier state. The concept of salutogenesis emphasizes health factors that promote and maintain good health rather than focusing solely on pathogenesis (Antonovsky, 1979; Mittelmark and Bauer, 2017).
Stress	Stress is a physical and emotional reaction that people experience as they encounter challenges in life. When a person is under stress, the body reacts by releasing hormones that produce the “fight-or-flight” response. Occasional stress is a normal coping mechanism; however, long-term stress (also called chronic stress) negatively impacts every organ system of the body: “fight or flight” response overdrive, elevated stress hormones, disrupted sleep, muscle tension, metabolic dysfunction, immune dysregulation, and inflammation (NIH, n.d.).
Resilience	The process that integrates multiple central and peripheral systems in response to stress and is therefore key to salutogenesis. The Trans-NIH Resilience Working Group defines resilience as “the capacity to resist, adapt to, recover, or grow from a challenge” (Brown et al., 2023).
Emotional well-being	A multi-dimensional composite that encompasses how positive an individual feels generally and about life overall. It includes both experiential features (emotional quality of momentary and everyday experiences) and reflective features (judgments about life satisfaction, sense of meaning and purpose, and ability to pursue goals that can include and extend beyond the self) (Park et al., 2023; Necka et al., 2022).

**Figure 1:**
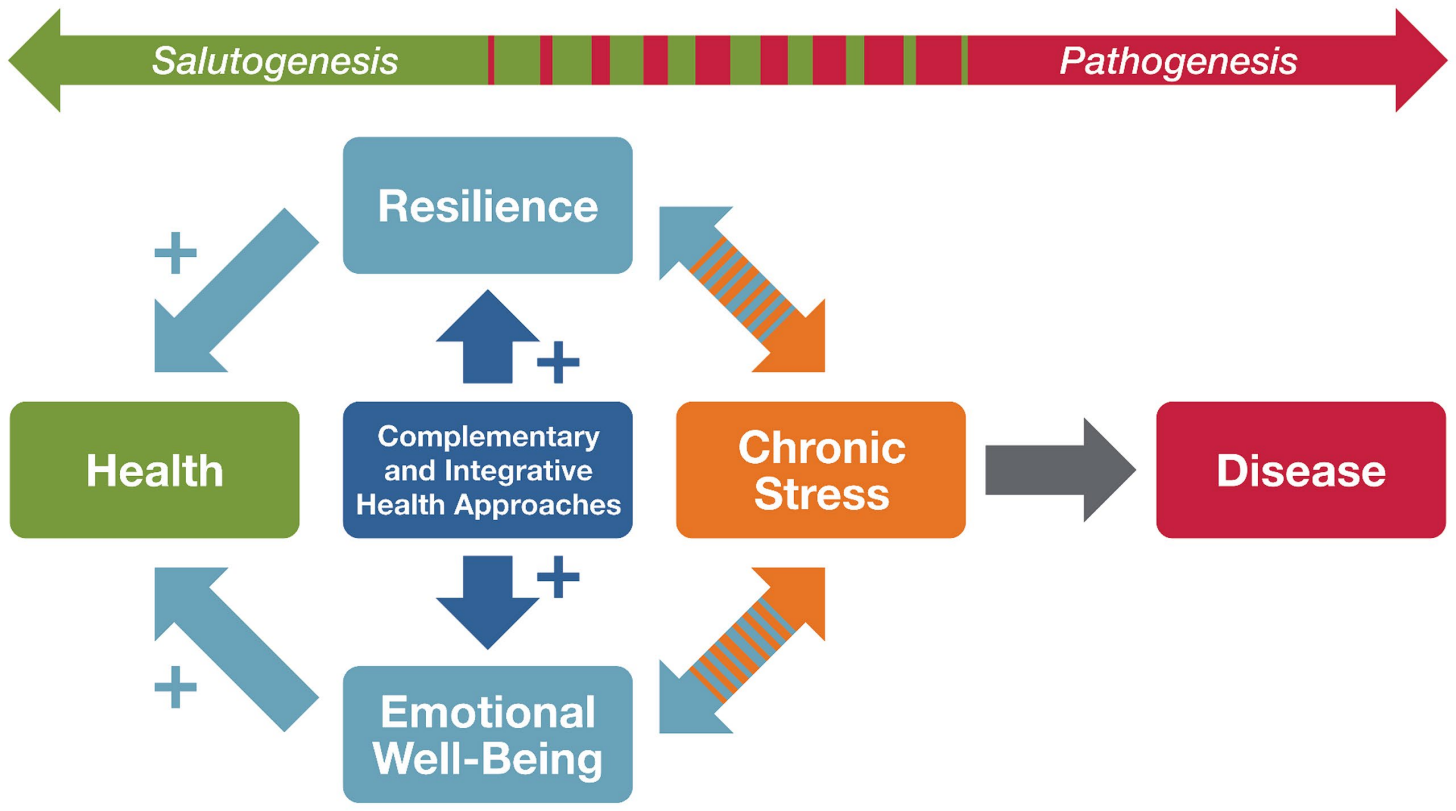
Salutogenic conceptual model of how complementary and integrative health approaches may buffer the negative effects of chronic stress on health by promoting resilience and emotional well-being.

Identifying factors and interventions to manage chronic stress, and rigorously understanding the salutary processes by which they exert their effects, has the potential to prevent multiple diseases and restore whole person health. We propose that two malleable factors have the potential to engage salutogenic processes in response to stress—resilience and EWB—both of which are multidimensional concepts. The goal of this Perspective is to propose that resilience and EWB are concepts that fit uniquely within a salutogenic model of health in regard to stress. We delineate through the available evidence that both concepts may engage salutogenic pathways and have the potential to restore health in the context of chronic stress (McEwen, 2017) (Figure 1). Further, we provide examples from the literature that demonstrate complementary and integrative health approaches, including mind and body interventions (e.g., meditation, yoga, tai chi) and natural products, that have the potential to move people along these pathways to restore health (Benson, 2019; NIH, 2020). We also present examples of research across multiple disciplines linking these constructs and propose a conceptual model for incorporating complementary and integrative health approaches to build resilience and EWB as modalities for improving health (Brown et al., 2023; Cathomas et al., 2019; Langevin et al., 2023).

## How resilience and emotional well-being may affect health in response to chronic stress

### The relationship between resilience and health

Empirical research has shown that such resilient responses to challenges may significantly affect an individual’s health status. Resistance refers to an individual’s ability to withstand stress without showing signs of distress or dysfunction. As demonstrated through studies on immune resilience, certain individuals can maintain robust immune function despite high-stress conditions such as SARS-CoV-2 infections (Carsetti et al., 2020). People with strong support networks also exhibit lower levels of stress-related illnesses, likely due to psychological resistance (Mann and Walker, 2022).

Recovery is the process of returning to a baseline level of functioning after experiencing stress. For example, the immune system may play an important role in recovery, where individuals with immune disorders often experience stress responses that can lead to poor emotional and behavioral health (Cooke, 2019). Others may recover only partially but adapt to a new level of functioning in response to pathogenic insult or stress, which may be considered as partial recovery or tolerance (Bergstrom et al., 2012).

Adaptation involves adjustments made by individuals in response to stressors, enhancing their ability to cope with future stress. Organisms may adapt to environmental stressors to improve survival and reproductive success through evolutionary adaptations (Bijlsma and Loeschcke, 2005). The concept of allostasis provides further insights into how the brain, gene expression, and epigenetics contribute to adaptation, allowing individuals to maintain stability through change (McEwen, 2016b).

Growth, including post-traumatic growth, refers to the positive psychological change experienced due to experiencing highly challenging circumstances. Growth can be physical in addition to psychological (Carver, 1998). Individuals with higher resilience levels tend to report better mental and physical health, likely due to the protective effects of resilience against stress and adversity (Ameli et al., 2018). People may also undergo positive psychological and physical changes through adaptive coping strategies and social support, leading to enhanced personal strength, improved relationships, and a greater appreciation for life, even if they experienced significant adversity (Lowe et al., 2022; Weir, 2020). Deeper understanding of the relationships between resilience and health, in particular the underlying mechanisms, may identify resilience responses that can be modified to enhance health outcomes.

### The relationship between emotional well-being and health

Positive emotions and more broadly EWB have been associated with better health (Boehm et al., 2020; Richman, 2005; Stewart-Brown, 1998), including improved recovery and survival from physical illness (Hernandez et al., 2018; Lamers et al., 2012; Levine et al., 2021). Over the past decade, research has begun to identify potential mechanisms underlying the relationship between EWB and health.

Affective science researchers have described the importance of emotion regulation (Thompson, 1994) for health (DeSteno et al., 2013). Neuroscientific investigations of emotion regulation and emotion processing have identified mechanisms related to autonomic, endocrine, and immune functioning. Functional neuroimaging studies of the emotion regulation strategy, cognitive reappraisal (Buhle et al., 2014), demonstrate reliable activation of multiple prefrontal cortical areas, including those implicated in cardiovascular functioning (Gianaros and Wager, 2015; Thayer et al., 2012). Cognitive reappraisal may even affect the connection between heart rate variability and telomere length (Shahane et al., 2020). A neuro-immune-affective framework (Lopez et al., 2018) in the context of EWB is supported by a large cross-sectional U.S. survey showing that the EWB constructs of positive affect and life satisfaction are significantly related to lower C-Reactive protein, an inflammatory biomarker (Ironson et al., 2018).

EWB may improve health across the lifespan through its relationship with social connection (Courtwright et al., 2020; Ermer and Proulx, 2019), with social connectedness potentially mediating positive emotional, behavioral, and psychological functioning (Mauss et al., 2011). The neuropeptide oxytocin may underlie this relationship and modulate various aspects of social behaviors (Arias Del Razo et al., 2022; Bales et al., 2013; Shamay-Tsoory and Abu-Akel, 2016). Studies of social deficits in rodents suggest another potential mechanism, activation of the stress-related hypothalamic–pituitary axis circuits, as isolated and lonely individuals show higher anxiety and hypervigilance to negative social stimuli (Matthews and Tye, 2019).

Emotion regulation strategies may differ with temporal and situational contexts (Gross, 2015) as well as age (Brockman et al., 2017), suggesting the biological mechanisms underlying emotion regulation, EWB, and health are likely not one-size-fits-all and require further mechanistic exploration in longitudinal contexts. More research is needed linking how the mechanisms underlying the relationship between EWB and health are affected by chronic stress. Furthermore, the definitions and measures used to capture EWB vary widely, so future research should aim to use harmonized measures grounded in a common EWB framework (e.g., the definition put forth by Park et al., 2023). Improved understanding of the biological and behavioral mechanisms linking EWB and health could identify potential therapeutic targets for complementary and integrative health approaches.

### How complementary and integrative health approaches may influence resilience and EWB to manage stress and promote health

In the context of whole person health, the National Center for Complementary and Integrative Health at NIH has categorized complementary and integrative health approaches according to their primary therapeutic inputs—nutritional, psychological, and physical categories that partially overlap with one another and with conventional therapeutics (e.g., pharmaceuticals). These approaches include mind and body practices, such as mindfulness meditation, yoga, and tai chi, and natural products, such as medicinal plants, probiotics, and dietary supplements (NIH, 2021). The use of these complementary and integrative approaches is becoming increasing popular in the United States (Clarke et al., 2018; Nahin et al., 2024). We propose that some complementary and integrative health approaches may be uniquely positioned, potentially though the mechanisms delineated in the previous section of this paper, to engage salutogenic pathways in transitioning from a chronically stressed state to one of improved health as a function of resilience and EWB.

Mind and body interventions such as mindfulness meditation, yoga, and tai chi have generated a strong evidence base for improving aspects of EWB, including life satisfaction, psychological well-being, and positive affect, particularly as measured by self-report in clinical populations (Garland et al., 2017; Goyal et al., 2014; Hendriks et al., 2017). Studies demonstrating that engagement of EWB by mind and body practices on salutary outcomes are few but emerging in clinical populations. In a sample of older adults with depression, 10 weeks of tai chi as an adjunct to standard pharmacotherapy produced greater health restorative effects as measured by higher rates of depression remission and greater immunity than an active control condition (Lavretsky et al., 2011). A recent study involving patients with opioid use disorder who received 8 weeks of Mindfulness-Oriented Recovery Enhancement—an intervention that integrates skills relevant to EWB (e.g., mindfulness, reappraisal, and savoring)—plus usual care had significantly less return to drug use, as well as reduced pain and depression scores, compared to usual care alone (Cooperman et al., 2024). Additional rigorously designed studies are needed to parse explicit associations among stress, EWB (measured as a multidimensional composite), salutogenesis, and the extent to which these factors are modulated by mind and body interventions.

There is growing evidence that mind and body interventions, like mindfulness meditation, improve the ability to mount resilient responses to stressors in physically and psychologically stressed individuals. Mindfulness is thought to mitigate stress pathways through deliberate nonjudgment and nonreactivity toward sensory and emotional experiences, possibly increasing personal distance or the ability to engage flexibility with stressors. In chronically stressed samples of unemployed (Lindsay et al., 2018) and obese adults (Daubenmier et al., 2019), mindfulness skills training was associated with greater peripheral resistance and recovery in response to laboratory stressors, when compared to active controls. At the neural level, U.S. Marines who underwent 8 weeks of mindfulness training during the highly stressful predeployment period displayed attenuated right anterior insula and anterior cingulate activity during an aversive interoceptive stimulation, possibly reflecting protective effects that reserve self-regulatory mechanisms normally depleted during chronic stress (Haase et al., 2016). However, in these studies, the extent to which these effects prevent downstream health consequences or engage restorative, salutogenic processes was untested. One way mind and body-based resilience could impart salutogenesis is through stabilizing telomere length (Conklin et al., 2019), which is shortened by chronic stress (Epel and Prather, 2018), thereby impairing immune capacity (Cohen et al., 2013). Additional research is needed to characterize patterns in response to stressors using innovative methods, measurement tools, and in larger sample sizes.

Data indicate that more than 83 percent of those who used natural products reported using them for general wellness or disease prevention (Stussman et al., 2015). It is well established that a diet rich in fruits and vegetables and lower in saturated and trans-fats promotes health and prevents disease, and that an unhealthy diet of excess calories, fat, and sugar can lead to many pathological processes that promote obesity, cardiovascular disease, diabetes, and cancer. Beyond the foods that individuals consume, more than 57 percent of U.S. adults over age 20 have taken a dietary supplement (e.g., botanicals, vitamins, omega-3 fatty acids, minerals and/or probiotics) in the last 30 days (Mishra et al., 2021). Some botanicals that may be relevant to the concept of salutogenesis have been described as having the ability to assist an organism in its response to stressors or to have the effect of normalizing function or enhancing mental or physical performance (Todorova et al., 2021). Several botanicals are hypothesized to have these properties, including *Panax ginseng, Eleuthrococcus senticosus, Rhodiola rosea, and Schisandra chinensis* (Todorova et al., 2021). Evidence to understand the mechanisms and clinical effects of extracts of these botanicals is limited but growing.

Comprehensive summaries of the evidence from basic, animal, and human studies on the anti-inflammatory, stress resilience, and endurance enhancement effects of botanicals are available in the literature (Anghelescu et al., 2018; Ivanova and Quintela, 2022; Speers et al., 2021; Todorova et al., 2021; Wróbel-Biedrawa and Podolak, 2024). Here, we describe the results of a few intriguing studies that demonstrate the potential of botanicals for stress-related conditions and the promotion of salutogenesis. One placebo-controlled-randomized trial examined the impact of a 28-day course of a combined product including magnesium, B vitamins, Rhodiola and green tea (L-theanine) on 100 chronically stressed healthy individuals. The study found a reduction in stress scores from baseline to day 28, significant reduction in sensitivity to cold pain, and a trend for lower sensitivity to warm pain (Noah et al., 2022). In addition to botanical extracts, researchers are also studying the effects of pre-and/or probiotics on stress resilience. For example, Westfall et al. (2021) found that a combination of pre-and probiotics (i.e., bioactive dietary polyphenol preparation including grape seed extract and grape extract, and *Latobacillus plantarum* and *Bifidobacterium longum*) improved resilience to a chronic unpredictable stress model in mice, which resulted in an attenuated impact on immune function and depressive and anxiety-like behaviors (Westfall et al., 2021). Most trials on these types of natural products are small or uncontrolled (Todorova et al., 2021). Overall, more research is needed to measure the feasibility, efficacy, and effectiveness of a wider range of interventions including multicomponent interventions across the lifespan and in diverse populations in the context of prevention, health promotion, resilience, EWB, and health restoration.

### Moderators of relationships between resilience, EWB, and health

Resilience and EWB and their potential influences on health restoration require adopting a whole person health orientation that considers multiple levels of complexity, including the intraindividual, interpersonal, community, and population levels. Genetic factors, coping strategies, social support, lifestyle factors such as nutrition and physical activity, as well as dispositional factors can all interact to foster resilience, EWB, and health restoration in the context of chronic stress (Vaccarino and Bremner, 2024). One concept with strong research support is pre-existing levels of reserve capacity, operationalized as the personal resources of an individual that confer protection against stress, including aggregate levels of optimism and sense of control, and factors such as social support (Matthews et al., 2008; Epel, 2020). Recent views further propose that effective routes to resilience and positive aspects of adjustment and well-being in the face of high stress requires the flexible use of resource capacity phenotypes, a concept referred to as regulatory capacity (Bonanno et al., 2023). How much this capacity is modifiable remains uncertain, but it is a promising avenue for future investigation.

Intraindividual and interpersonal factors, referred to as social determinants of health (SDoH), encompassing economic, social, environmental, and psychosocial factors have profound influences on health. Central to the connection between SDoH and pathogenesis is excessive threat that contributes to repeated instantiation of the stress response and allostatic load through mechanisms such as inflammation and cellular aging (Brisson et al., 2020; Powell-Wiley et al., 2022). Individuals vulnerable to negative SDoH (e.g., poor housing, violent neighborhoods, structural racism) experience significant challenges in culminating the needed resources in which resilience and EWB can manifest (Matthews et al., 2008). A more nuanced, multi-level understanding of the conditions in which resilience and EWB can promote salutogenesis of the whole person has great potential to lead to important and sustained benefits in population health.

## Discussion

With chronic stress and poor physical and mental health on the rise, identifying ways to buffer the negative effects of chronic stress is of urgent public health importance. If prolonged, severe, or during important developmental periods, chronic stress can also negatively impact an individual’s EWB and ability to adapt to future stressors. However, individuals exhibiting greater resilience and EWB may fare better under chronic stress. The extant literature suggests there are neurobiological, physiological, and behavioral processes that may underlie the positive associations between resilience, EWB, and health. In this paper we posit a framework of how complementary and integrative health approaches, such as mind and body interventions and natural products, might mitigate the effects of chronic stress by engaging, and possibly enhancing, salutogenic processes.

However, more empirical research is needed with important considerations based on some identified gaps and limitations. The research to date has explored parts of the framework but has not tested the main hypotheses proposed herein. For example, longitudinal neuroimaging studies of mindfulness practices in stressed populations have not also measured health outcomes or how mindfulness might also engage restorative processes. Compared to research on mind and body interventions like mindfulness and yoga, there are far fewer studies exploring the mechanisms and clinical effects of diet and natural products in the context of resilience, EWB, and health. To support the reproducibility and translation of future health promotion research, studies would benefit from harmonizing the definition and measurements used for constructs like resilience and EWB. Finally, likely moderators of salutogenic processes such as individual characteristics and factors (e.g., genetics, age, gender, social support, lifestyle factors) should be considered and integrated into the study design. We envisage rigorous longitudinal mechanistic and clinical research to inform the optimization of existing, and development of new complementary and integrative health approaches that promote resilience, EWB, and health in the face of rising chronic stress.

## Data Availability

The original contributions presented in the study are included in the article/supplementary material, further inquiries can be directed to the corresponding author.
